# Environmental Adaptability and Organic Pollutant Degradation Capacity of a Novel *Rhodococcus* Species Derived from Soil in the Uninhabited Area of the Qinghai-Tibet Plateau

**DOI:** 10.3390/microorganisms10101935

**Published:** 2022-09-29

**Authors:** Jiao Huang, Guomin Ai, Ning Liu, Ying Huang

**Affiliations:** 1 State Key Laboratory of Microbial Resources, Institute of Microbiology, Chinese Academy of Sciences, Beijing 100101, China; 2 College of Life Sciences, University of Chinese Academy of Sciences, Beijing 100049, China

**Keywords:** *Rhodococcus tibetensis* FXJ9.536, adaptation strategy, Qinghai-Tibet Plateau, *p*-nitrophenol (4-NP), malathion, degradation

## Abstract

The Qinghai-Tibet Plateau (QTP) is known for extreme natural environments and, surprisingly, has been reported to contain widespread organic pollutants. *Rhodococcus* can survive a variety of extreme environments and degrade many organic contaminants. Here, we isolated a *Rhodococcus* strain (FXJ9.536 = CGMCC 4.7853) from a soil sample collected in the QTP. Phylogenomic analysis indicated that the strain represents a novel *Rhodococcus* species, for which the name *Rhodococcus tibetensis* sp. nov. is proposed. Interestingly, *R. tibetensis* FXJ9.536 maintained a fast growth rate and degraded 6.2% of *p*-nitrophenol (4-NP) and 50.0% of malathion even at 10 °C. It could degrade 53.6% of 4-NP and 99.9% of malathion at a moderate temperature. The genome of *R. tibetensis* FXJ9.536 contains 4-hydroxyphenylacetate 3-monoxygenase and carboxylesterase genes, which are likely associated with the degradation of 4-NP and malathion, respectively. Further genomic analysis revealed that the strain might employ multiple strategies to adapt to the harsh QTP environment. These include synthesizing cold shock proteins, compatible solutes, secondary metabolites, and storage compounds, utilizing inorganic compounds as energy and nutrition sources, as well as degrading a range of organic pollutants. Overall, our study reveals the potential of a QTP-derived new actinobacterial species for environmental adaptation and remediation in cold regions.

## 1. Introduction

Microbes living in extreme environments have evolved diverse survival strategies to cope with harsh environmental conditions. For example, to survive the cold environment, they can alter the fluidity of cell membranes [1], secret cold-adaptable enzymes [2], accumulate polyunsaturated fatty acids [3], and produce bioactive substances such as trehalose, glycerol, lipase, ectoine, carotenoid, hopene, and pigments [4,5]. The genus *Rhodococcus*, which belongs to the phylum *Actinobacteria*, can survive and thrive in a variety of extreme environments [6]. Some species of *Rhodococcus* isolated from alpine areas, the Arctic, and the Antarctic have the ability to adapt to cold environments [7,8,9,10]. Rhodococci in cold environments use multiple strategies, such as cold shock proteins (CSPs), storage compounds (glycogen, polyhydroxyalkanoates (PHAs), and triacylglycerols (TAGs), etc.), and other factors related to membrane/cell wall alteration, to be suited to the surroundings [9,11,12,13,14]. *Rhodococcus* strains also showed amazing adaptability to habitats rich in organic pollutants, because of their degradation ability to a wide variety of organic contaminants such as polychlorinated biphenyls (PCBs), polycyclic aromatic hydrocarbons (PAHs), and pharmaceutical pollutants [7,15,16,17]. These characteristics make *Rhodococcus* a very promising bioremediation tool. In addition, over the last two decades, a few studies have revealed that *Rhodococcus* is a good source of natural products, which may also contribute to its environmental adaptation [3].

In recent decades, the rapid growth of environmental pollution has become a global problem threatening human life and health. Nitroaromatics are widely used as synthetic intermediates in the manufacture of pharmaceuticals, dyes, plastics, explosives, and industrial solvents, causing inevitable accumulation and pollution of nitroaromatics in environments [18,19]. *P*-nitrophenol (4-NP) is one of the most extensively used nitroaromatics and one of the pervasive pollutants in the environment [20,21,22]. As reported, 4-NP shows strong toxicity and mutagenic potential and is refractory in the environment, posing a threat to a variety of ecosystems [23]. Many microorganisms capable of degrading 4-NP have been isolated, and among them, the degrading strains of *Rhodococcus* were mostly derived from habitats contaminated with aromatic hydrocarbons and petroleum [16]. The degradation pathway in *Rhodococcus* *imtechensis* RKJ300 has been reported, in which 4-NP might be converted to maleylacetate via four putative enzymes: 4-NP monooxygenase, ring hydroxylating dioxygenase, hydroquinone dioxygenase, and hydrolase [24].

Organophosphate (OP) pollution is also a serious environmental problem owing to its wide use as pesticide in agriculture [25,26]. Among OPs, malathion has been used worldwide mainly to control weeds, diseases, and pests in agriculture and horticulture [27,28]. However, malathion is fatal to some beneficial insects, fishes, and freshwater invertebrates; even low doses of malathion can affect organisms adversely [29]. Natural degradation of malathion in environments depends on temperature and pH. When the ambient temperature is below 25 °C, malathion is very stable with a photolysis half-life above five months [30]. Microbial degradation is an important way to cope with malathion contamination for the degradation process can be greatly accelerated with the help of microorganisms [30]. To date, several bacteria and fungi have been reported to degrade malathion, using different pathways involving hydrolases, esterases, phosphatases, and oxidoreductases [27,30,31,32,33]. *Rhodococcus rhodochrous* also shows malathion degradation activity, but the related genes and catabolic pathway have not been illuminated [34].

The Qinghai-Tibet Plateau (QTP) is the largest (3.0 million km^2^) and highest (average altitude 4500 m) plateau on Earth, known as “the roof of the world” or “the third pole” [35]. The high altitude endows the QTP’s unique and extreme environmental characteristics: low annual average temperature, little precipitation, low humidity, oligotrophy, and prolonged UV radiation [36,37]. Although the QTP is often regarded as a “clean” region minimally disturbed by human activities, recent studies have provided surprising facts that organic pollutants, such as PCBs and PAHs, widely exist in the biota and environmental media of the QTP [38]. In this study, a novel strain, FXJ9.536, was isolated from soil in the uninhabited area of the QTP and identified as representing a new species of *Rhodococcus* by genome-based phylogenetic analysis. Its growth traits and degradation activities towards 4-NP and malathion at different temperatures were investigated. Corresponding genetic underpinnings were also discussed according to bioinformatics analysis of its genome. The results suggest that this new species probably uses multiple strategies to adapt to the QTP environment and degrade organic pollutants.

## 2. Material and Methods

### 2.1. Sample Collection and Strain Isolation

The sample was collected from the surface layer (5 to 20 cm) soil in the uninhabited area of the QTP (89°6′46.008″ E, 34°56′22.004″ N, 5020 m altitude), western PR China, in April 2017. The diluted soil suspension was inoculated on GN agar (soluble starch 20.0 g, KNO_3_ 1.0 g, K_2_HPO_4_ 0.5 g, MgSO_4_·7H_2_O 0.5 g, NaCl 0.5 g, FeSO_4_·7H_2_O 0.01 g, agar 15.0 g, ddH_2_O 1000 mL, pH 7.0–7.2). The isolation plates were incubated for 2 weeks at 28 °C, and selected colonies were further purified on GYM agar (yeast extract 4.0 g, glucose 4.0 g, malt extract 10 g, agar 15.0 g, ddH_2_O 1000 mL, pH 7.0–7.2).

### 2.2. Growth Assay

Cells of strain FXJ9.536, cultured at 28 °C, 200 rpm overnight in LB medium (tryptone 10.0 g, yeast extract 5.0 g, NaCl 10.0 g, pH 7.0–7.2), were centrifuged at 5000 rpm for 10 min. The cell pellet was washed twice with and resuspended in sterile LB medium to OD_600_ = 0.8. Then, 100 μL LB medium and 5 μL resuspended inoculum were added to each well of 96-well plates, and the plates were incubated at 10 °C or 28 °C, 200 rpm. LB medium without inoculation was also run as negative controls at both temperatures. OD_600_ of the plate wells was periodically detected by a microplate reader (BioTek, Synergy H4) to draw the growth curve. Three parallel microplates were set for each temperature.

### 2.3. 4-NP Degradation Activity Assay

Cells of strain FXJ9.536 were resuspended in sterile MSM medium [(NH_4_)_2_SO_4_ 2.0 g, MgSO_4_·7H_2_O 0.2 g, CaCl_2_·2H_2_O 0.01 g, FeSO_4_·7H_2_O 0.001 g, Na_2_HPO_4_·12H_2_O, 1.5 g, KH_2_PO_4_ 1.5 g, ddH_2_O 1000 mL; pH 7.2 ± 0.2] to OD_600_ = 0.8. Then, 1 mL resuspended inoculum was inoculated into 20 mL MSM medium supplemented with 5 g/L glucose (as a co-substrate to improve 4-NP degradation) [39] and 100 μL 4-NP solution (20 mg/mL) in a 100-mL Erlenmeyer flask, in which the final concentration of 4-NP was 100 mg/L. The flasks were incubated in the dark at 10 °C or 28 °C, 200 rpm for 3 days. The fermentation was conducted in triplicate. The control is a sterile MSM medium containing a final concentration of 100 mg/L 4-NP. After that, the concentration of 4-NP in the culture supernatant was analyzed by high-performance liquid chromatography (HPLC) using a Shimadzu SPD-M20A HPLC system equipped with Athena C18 column (4.6 × 150 mm, 5 μm particles size, CNW), following previous procedures [39]. The residual 4-NP and its degradation intermediates were identified by Liquid Chromatography-Mass Spectrometry (LC-MS) in a negative mode using an Agilent 1260/6460 Triple quadrupole LC/MS system. The analytic column was ZORBAX SB-Aq (Agilent, 2.1 × 100 mm, 3.5 μm). A mixture of 10% acetonitrile and 0.1% formic acid (*v*/*v*) in water under an isocratic elution program was used as the mobile phase. The flow rate was 0.25 mL/min, and the column temperature was 30 °C. A fragmentor voltage of 125 V, a capillary voltage of 3.5 kV, and an atomizing gas pressure of 35 psi were used. The flow rate of drying gas was 12 mL/min and the temperature for solvent removal was 350 °C. The LC-MS data were acquired and analyzed using a MassHunter Workstation Software (Version B.04.00, Agilent, Santa Clara, CA, USA).

### 2.4. Malathion Degradation Activity Assay

Cells of strain FXJ9.536 were resuspended in sterile BM medium (K_2_HPO_4_ 1.0 g, MgSO_4_·7H_2_O 1.0 g, (NH_4_)_2_SO_4_ 2.0 g, NaCl 1.0 g, CaCO_3_ 2.0 g, ddH_2_O 1000 mL, pH 6.4–6.8) to OD_600_ = 0.8. Then, 0.5 mL resuspended inoculum was inoculated into a 10 mL BM medium supplemented with 0.5 g/L yeast extract, 0.5 g/L glucose (both as co-substrates to improve malathion degradation) [27], and 50 μL malathion solution (20 mg/mL) in a 50-mL erlenmeyer flask, in which the final concentration of malathion was 100 mg/L. The flasks were incubated in the dark at 10 °C or 24 °C, 200 rpm for 7 days. The fermentation was conducted in triplicate. Sterile malathion-BM medium was also run in parallel as acontrol. After fermentation, the residual malathion in broth was extracted by adding an equal volume of n-hexane. After vigorous shaking for 5 min, the organic layer was separated and dehydrated by passing through anhydrous Na_2_SO_4_. The extracted malathion was analyzed by gas chromatography-mass spectrometry (GC-MS) using Agilent 7200 Q-TOF GC/MS as previously described [40].

### 2.5. Genome Sequencing, Annotation and Bioinformatics Analyses

The genomic DNA of strain FXJ9.536 was extracted following the method proposed by Chun and Goodfellow [41] and sequenced using Illumina NovaSeq PE150 apparatus. Briefly, 1 μg DNA sample was fragmented by sonication to a size of 350 bp, then DNA fragments were end-polished, A-tailed, and ligated with the full-length adaptor for Illumina sequencing. The read quality was analyzed by FastQC (www.bioinformatics.babraham.ac.uk/projects/fastqc/ (accessed on 22 February 2021)). The de novo assembly of the genome was performed using SPAdes [42]. The completeness and contamination of the genome were assessed using CheckM (http://ecogenomics.githu.io/checkm/ (accessed on 26 June 2021)) [43]. The average nucleotide identity (ANI) between the genome of strain FXJ9.536 and other available genomes of *Rhodococcus* (Table S1) were analyzed using FastANI [44] based on blastn (ANIb). Annotation of the FXJ9.536 genome was carried out using RAST (https://rast.nmpdr.org/ (accessed on 26 June 2021)) [45]. The secondary metabolite biosynthetic gene clusters (sm-BGCs) were analyzed using antiSMASH version 5.0 [46].

Genes involved in 4-NP and malathion degradation in the genome of strain FXJ9.536 and 59 reference genomes of *Rhodococcus* spp. (Table S1) were detected using local BlastP. Amino acid (AA) sequences of the 4-NP hydroxylase component A derived from *Rhodococcus* sp. PN1 (GenBank No. BAB86378) and carboxylesterase (EC 3.1.1.1) derived from *Alicyclobacillus tengchongensis* D-1 (GenBank No. AGG09662), which were reported to be responsible for degrading 4-NP [47] and malathion [48], respectively, were set as query sequences for searching homologous sequences from the genomes. The cutoff value of the AA sequence similarity was set as 30%.

### 2.6. Phylogenetic Analyses

The phylogenetic trees based on 16S rRNA gene sequences and amino acid sequences of predicted 4-NP and malathion degradation genes, were reconstructed using the maximum-likelihood (ML) [49] and neighbor-joining (NJ) [50] tree-making methods implemented in MEGA-X [51]. The tree topology was evaluated by bootstrap analysis [52] following 1000 resamplings. A phylogenomic tree based on the core genes of FXJ9.536 and the reference *Rhodococcus* genomes was generated using the BPGA pipeline [53].

## 3. Results and Discussion

### 3.1. Identification of Rhodococcus tibetensis sp. nov.

**Figure 1:**
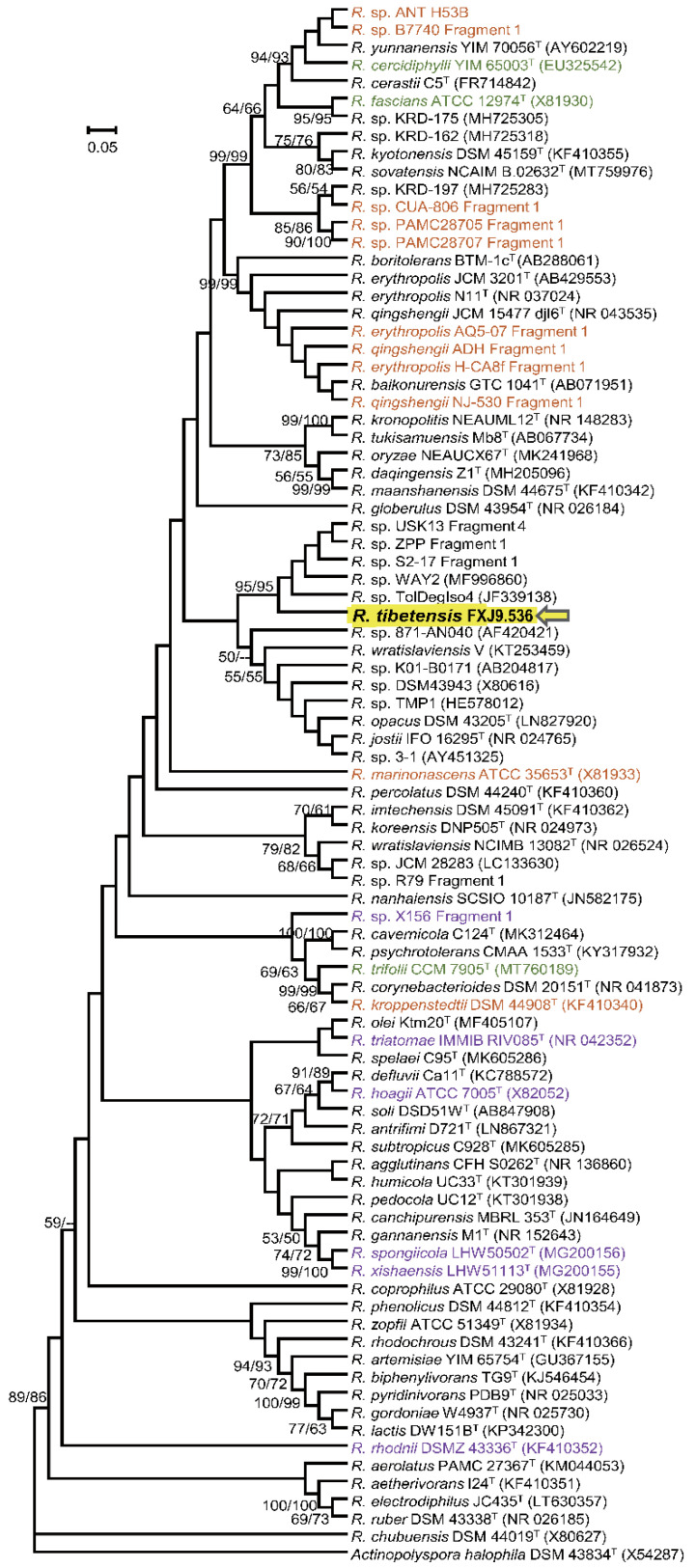
Maximum-likelihood phylogenetic tree based on 16S rRNA gene sequences of *Rhodococcus* spp. *Actinopolyspora halophila* DSM 43834^T^ (X54287) was used as an outgroup. Strains derived from plants, animals, and extreme environments (marine sediments, alpine regions, and polar regions), are highlighted in green, purple, and orange, respectively. Only bootstrap values above 50% are given. Bar, 0.05 substitutions per site.

Strain FXJ9.536 produced buff colonies after growing on GYM agar for 7 days at 28 °C and 10 °C, and white colonies at 4 °C; and the reverse side of the colonies was deep orange to pale orange (Figure S1). Neither soluble pigments nor melanin were observed. The strain shared high 16S rRNA sequence similarity with strains *Rhodococcus opacus* DSM 43205^T^ (99.34%), *Rhodococcus wratislaviensis* V (99.19%), and *Rhodococcus jostii* IFO 16295^T^ (98.46%). It showed even higher 16S rRNA sequence similarity (>99.7%) with five unnamed strains, *R.* sp. USK13, *R.* sp. ZPP, *R.* sp. S2-17, *R.* sp. WAY2, and *R.* sp. TolDegIso4. In the phylogenetic tree of the 16S rRNA gene, FXJ9.536 was clustered with the above-mentioned five unnamed strains in a small subclade, which was diverged from another subclade containing the above-mentioned three named strains (Figure 1). These results clearly indicate that strain FXJ9.536 belong to the genus *Rhodococcus*.

**Figure 2:**
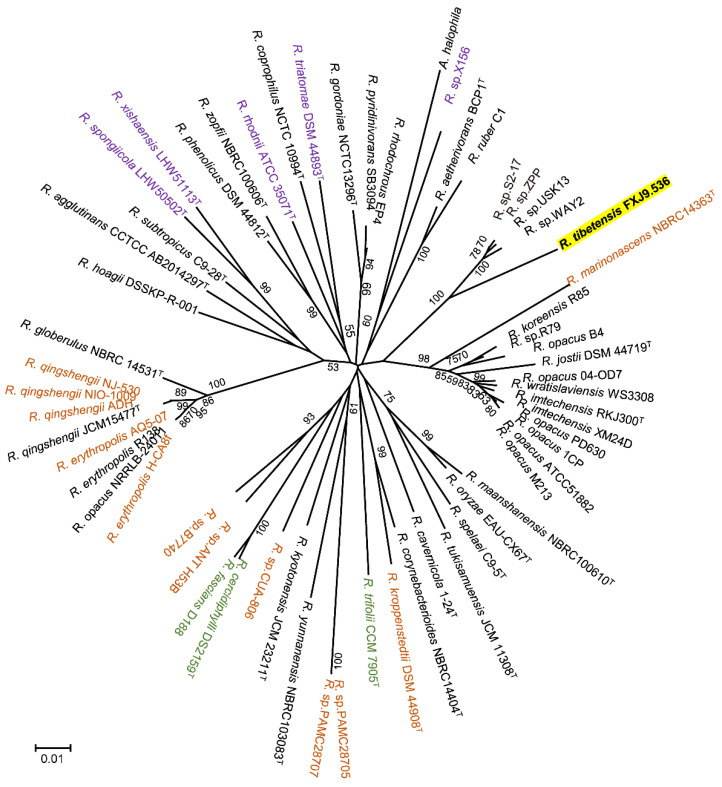
Maximum-likelihood phylogenomic tree generated from concatenated 450 core genes of *R. tibetensis* FXJ9.536 and 59 related strains of the genus *Rhodococcus*. *A. halophila* DSM 43834^T^ was used as an outgroup. Strains derived from plants, animals, and extreme environments (marine sediments, alpine regions, and polar regions) are highlighted in green, purple, and orange, respectively. Only bootstrap values above 50% are given. Bar, 0.01 substitutions per site.

The genome sequencing of strain FXJ9.536 generated approximately 1 Gb of clean data (~100-fold genome coverage), which were assembled into 63 scaffolds of 5,814,613 bp in total length with a G+C content of 65.84 mol%. The genomic sequence of strain FXJ9.536 had ≥99.87% completeness with ≤0.13% contamination. The genome-wide ANI values between strain FXJ9.536 and 59 other *Rhodococcus* strains (including several named strains of the closest species *R. opacus* and the above-mentioned closest unnamed strains) ranged between 76.06 and 83.58% (Table S2). These values are well below the ANI threshold of 95–96% for prokaryotic species demarcation [54], suggesting that strain FXJ9.536 represents a novel species of *Rhodococcus*. Moreover, a phylogenomic tree based on 450 core genes of *Rhodococcus* showed that strain FXJ9.536 was separated from the other members of *Rhodococcus*, stably located at the periphery of the clade formed by the unnamed strains with the closest 16S rRNA genes (*R.* sp. USK13, *R.* sp. ZPP, *R.* sp. S2-17, and *R.* sp. WAY2) (Figure 2). Therefore, the phylogenomic data support that strain FXJ9.536 belongs to a novel species of the genus *Rhodococcus*. Based on the ANI and phylogenomic evidence, we propose that strain FXJ9.536 represents a novel species, with the name *Rhodococcus tibetensis* sp. nov.

### 3.2. Growth of R. tibetensis FXJ9.536 at 28 °C and 10 °C

**Figure 3:**
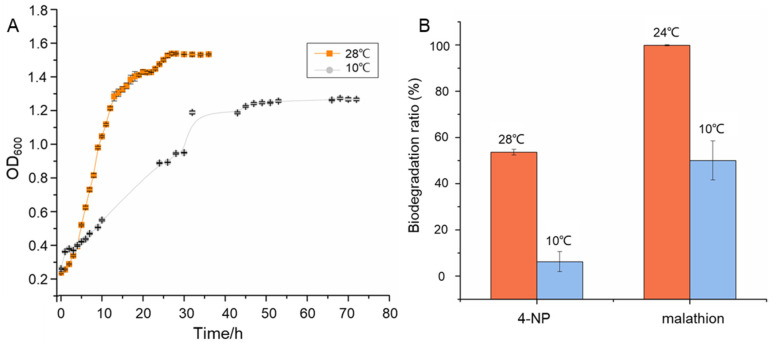
Growth (**A**) and biodegradation ability (**B**) of *R. tibetensis* FXJ9.536 at different temperatures. Error bars show the mean  ±  standard deviation of three independent experiments.

As the QTP has the characteristics of perennial low temperature and transient normal temperature, the growth of *R. tibetensis* FXJ9.536 at low temperature (10 °C) and normal temperature (28 °C) was examined. As shown in Figure 3A, although the growth rate at 10 °C is lower than that at 28 °C, the OD_600_ value of the 10 °C culture also exceeded 1.2 after the exponential growth phase (36 h). This result indicates that strain FXJ9.536 can adapt to low temperatures, which may support its survival in the QTP environment. Likewise, *Rhodococcus* sp. JG3, a strain isolated from Antarctic Dry Valley permafrost, also displays growth even at a subzero temperature [9].

### 3.3. 4-NP Degradation Activity of R. tibetensis FXJ9.536 and Bioinformatics Analysis of the Putative Key Catabolic Genes

It is reported that the natural environment of the QTP contains organic pollutants and *Rhodococcus* can degrade such pollutants [16,38]. To assess the potential of *R. tibetensis* FXJ9.536 for pollutant degradation, we investigated its 4-NP degradation activity at both 28 °C and 10 °C, two temperatures at which the strain grew well (Figure 3A). The result showed that *R. tibetensis* FXJ9.536 exhibited strong degradation activity at 28 °C, with a degradation ratio of 53.62%, and weak degradation activity at 10 °C, with a degradation ratio of 6.24% (Figure 3B and Figure S2). According to the HPLC and MS analyses of degradation residuals (Figure S2A–F), degradation intermediates 4-nitrocatechol and 1,2,4-benzenetriol were found. Consequently, we propose that the 4-NP degradation pathway in strain FXJ9.536 is via hydroxyquinol (1,2,4-bentenetriol) (Figure S2G), one of the two general pathways to convert 4-NP to maleylacetate [39]. Further genome scan revealed that two genes in *R. tibetensis* FXJ9.536, *peg.5460* and *peg.3921*, might be responsible for 4-NP degradation. Both the two genes encode putative 4-hydroxyphenylacetate 3-monoxygenases (EC 1.14.14.9), which shared 64.33% and 64.13% AA sequence similarities, respectively, with 4-NP hydroxylase component A, a reported enzyme in the 4-NP degradation pathway of *Rhodococcus* sp. PN1 (Table S3) [47]. The low degradation ratio of strain FXJ9.536 at 10 °C was probably due to the limited total amount of enzymes produced by the strain and/or the low enzyme activity at low temperature.

**Figure 4:**
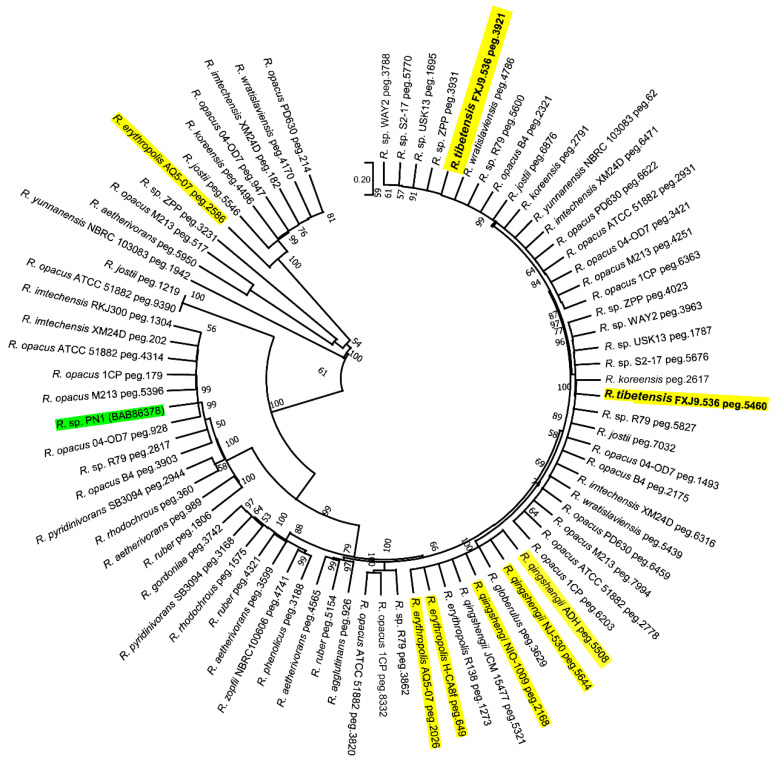
Maximum-likelihood phylogenetic tree based on amino acid sequences of the putative 4-hydroxyphenylacetate 3-monooxygenase genes in *Rhodococcus*. Sequences from cold environments and *Rhodococcus* sp. PN1 (4-NP hydroxylase component A) was highlighted in yellow and green, respectively. Only bootstrap values above 50% are given. Bar, 0.20 substitutions per site.

Many *Rhodococcus* strains have been reported to degrade 4-NP at normal temperature (28–30 °C). Thus, we analyzed the distribution of the 4-hydroxyphenylacetate 3-monoxygenase gene, which may relate to 4-NP degradation, in *Rhodococcus* genomes used in this study (Table S1). The result showed that 54.10% (33/60) of the genomes may harbor this gene, including the genomes from *Rhodococcus erythropolis* and *Rhodococcus qingshengii* strains that were derived from cold habitats. Furthermore, ML and NJ phylogenetic trees based on AA sequences of the monooxygenase genes were constructed. Both trees placed Peg.5460 and Peg.3921 together with some other sequences in two stable branches loosely related to the recognized 4-NP hydroxylase component A for 4-NP biodegradation (Figure 4 and Figure S3). A number of *Rhodococcus* strains also contain multiple putative 4-hydroxyphenylacetate 3-monoxygenases located in different phylogenetic branches, as exemplified by *R. erythropolis* AQ5-07 from Antarctic soil. The data above illustrate that degradation of 4-NP may be also common in *Rhodococcus* strains inhabiting cold environments.

### 3.4. Malathion Degradation Activity of R. tibetensis FXJ9.536 and Bioinformatics Analysis of the Putative Key Catabolic Genes

The degradation activity of *R. tibetensis* FXJ9.536 towards malathion was evaluated under 24 °C and 10 °C, as malathion tent to be hydrolyzed or photolyzed when the temperature is higher than 25 °C [30]. The results showed that strain FXJ9.536 exhibited very strong degradation activity to malathion at 24 °C, with a degradation ratio of 99.85%, and also strong activity at 10 °C, with a degradation ratio of 50% (Figure 3B and Figure S4). Then, genes that may participate in malathion degradation were detected. Gene *peg.1365*, which encodes a putative carboxylesterase, shared a 31.5% similarity with the AA sequence of carboxylesterase involved in malathion degradation derived from *Alicyclobacillus tengchongensis* D-1 [48] (Table S3). The carboxylesterase family is a group of esterases catalyzing the cleavage of carboxylic ester linkages [55]. Hence, we suspect that *R. tibetensis* FXJ9.536 also uses carboxylesterase to degrade malathion. Many microorganisms are capable of degrading malathion, and some species even use malathion as the sole carbon source [32,33,42]. However, only one *Rhodococcus* species has been reported to degrade malathion [34]. Our finding provides another example of the degradation of malathion by *Rhodococcus.* Because of the persistence and stability of malathion in natural environments, the degradation activity of strain FXJ9.536 at low temperatures makes it a potent bioremediation tool for malathion contamination in cold environments.

**Figure 5:**
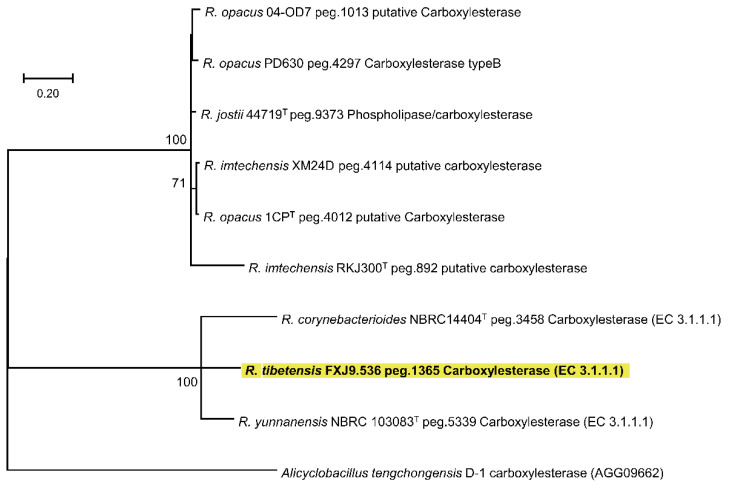
Maximum-likelihood phylogenetic tree based on amino acid sequences of the putative carboxylesterase genes. The carboxylesterase sequence associated with malathion degradation in *Alicyclobacillus tengchongensis* D-1 was used as an outgroup. Only bootstrap values above 50% are given. Bar, 0.20 substitutions per site.

To study whether the degradation of malathion is universal in the genus of *Rhodococcus*, we analyzed the distribution of the carboxylesterase (EC 3.1.1.1) gene in another 59 *Rhodococcus* genomes. The result showed that only eight (13.6%) genomes may harbor this gene. Alignment of the translated AA sequences revealed that Peg.1365 of *R. tibetensis* FXJ9.536 showed the highest similarity with Peg.5339 from *Rhodococcus yunnanensis* NBRC 103083 (66.73%) and Peg.3458 from *Rhodococcus corynebacterioides* NBRC14404 (60.20%). In the ML and NJ phylogenetic trees of carboxylesterase, the above three proteins also clustered together and diverged from putative carboxylesterases of another six *Rhodococcus* spp. (Figure 5 and Figure S5). The *Rhodococcus* sequences form two branches loosely related to the carboxylesterase sequence of *Alicyclobacillus tengchongensis* D-1 (Figure 5 and Figure S5). These data reveal that the degradation of malathion is not common in *Rhodococcus* and the degrading process may be executed by two types of carboxylesterases.

### 3.5. Bioinformatics Analysis of Adaptation Strategies of R. tibetensis FXJ9.536 to the Extreme Environment

It is well known that the annual mean temperature of the QTP is extremely low [35,36,37], and various kinds of stresses, such as osmotic stress and starvation, can be triggered by cold conditions [6]. To survive in such a cold environment, *R. tibetensis* FXJ9.536 may have evolved multiple adaptation strategies besides maintaining adequate growth and keeping considerably effective degradation activity at low temperatures (Figure 3). Bioinformatics analysis provides genomic evidence for this hypothesis.

**Figure 6:**
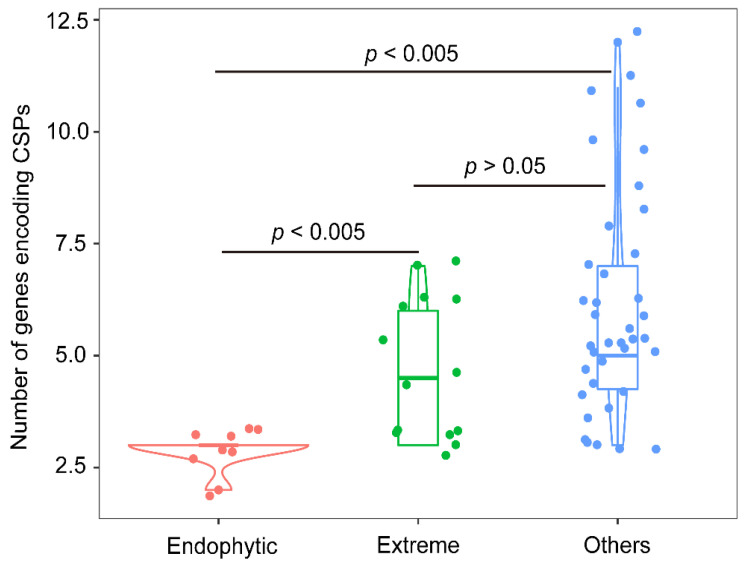
Distribution of cold shock protein (CSP)-coding genes in the genomes of *Rhodococcus* strains from different environments. The violin with a box plot shows the median and interquartile range, and the width of the violin represents the density distribution of the number of CSP-coding genes. *p* values were determined by the two-tailed Wilcoxon test. The number of *Rhodococcus* strains from endophytic, extreme, and other environments are 8, 13, and 39, respectively.

Firstly, cold shock proteins (CSPs), which can bind to DNA/RNA and are involved in the regulation of transcription and translation processes at low temperatures, maybe the foremost universal mechanism for cold adaptation [9,56]. In *Rhodococcus* sp. JG3, and CSPs have been putatively linked to the ability of growth at subzero temperatures [9]. The genome of *R. tibetensis* FXJ9.536 contains seven CSP-coding genes, six for CspA and one for CspC (Table S4). In *E. coli* and many other microorganisms, CspA is the main cold-induced CSP, and CspC is often involved in the regulation of several important physiological processes rather than cold shock [57,58,59]. Accordingly, the CspA genes may be more important than the CspC gene in strain FXJ9.536 for cold adaptation. To further understand the function of CSPs, a total of 319 CSP-coding genes were retrieved from the genomes of FXJ9.536 and 59 reference rhodococci. The number of CSP-coding genes in the individual genome ranged between 2 and 12 (Table S5). Specifically, the gene number in strains from endophytic (animals and plants), extreme (marine sediments, alpine regions, and polar regions), and other open environments were 2–3, 3–7, and 3–12, respectively (Table S5, Figure 6). Strains derived from extreme and other free-living environments contain significantly more CSP-coding genes than the symbiotic strains (Figure 6; *p* < 0.005), probably for the sake of habitat adaptation and wide distribution [56,60]. Therefore, the relatively high copy number of CSP genes in *R. tibetensis* FXJ9.536 may also contribute to its cold adaptation. Besides, the 60 *Rhodococcus* genomes each contain a single copy of the CspC gene, implying its important housekeeping function rather than cold adaptation.

Secondly, the anabolic genes of compatible solutes, such as trehalose, ectoine, and glycine betaine, were also found in the genome of *R. tibetensis* FXJ9.536 (Table S4). Trehalose, a naturally occurring disaccharide, which is thought to prevent the denaturation and aggregation of proteins during temperature downshift, may also stabilize cell membranes, whose fluidity decreases due to cold stress [61]. Three known trehalose biosynthetic pathways were found from the FXJ9.536 genome: 1, Trehalose-6-phosphate phosphatase (TPP, EC 3.1.3.12); 2, maltooligosyltrehalose synthase (MTSase, EC 5.4.99.15) and maltooligosyltrehalose trehalohydrolase (MTHase, EC 3.2.1.141); 3, trehalose synthase (TS, EC 5.4.99.16) and another trehalose synthase (TSⅡ) [62] (Table S4). The high number of genes associated with trehalose synthesis provides an important clue for the adaptation to cold and arid environments [61,62]. In view of the above observation, genes associated with the trehalose biosynthetic pathway were also investigated in 59 other reference genomes of *Rhodococcus*. All these genomes contain genes associated with the first two trehalose biosynthetic pathways; 25 genomes contain TS; 27 genomes contain TSⅡ, and 17 genomes carry both TS and TSⅡ genes (Table S5). The wide distribution of genes involved in trehalose biosynthesis among the 60 *Rhodococcus* genomes indicates that trehalose may be extremely important to the global environmental adaptation of *Rhodococcus*. It is reported that ectoine and glycine betaines are efficient cold adaptation strategies in *Vibrio anguillarum* and *Listeria monocytogenes*, respectively [63,64]. Therefore, the two compatible solutes may also contribute to the cold adaptation of strain FXJ9.536.

Thirdly, the genes for the assimilation of small inorganic molecules such as H_2_, CO, and CO_2_ were also found in the FXJ9.536 genome (Table S4). The [NiFe] hydrogenase system (Figure S6, Table S4), which is proposed to be responsible for high-affinity H_2_ oxidation to obtain energy in several actinobacteria groups, plays vital roles in surviving extreme environments especially oligotrophic conditions. This system provides cross-protection against desiccation, cold, and other environmental stresses [65,66]. Hence, the gene cluster of [NiFe] hydrogenase in *R. tibetensis* FXJ9.536 may play an important role in its adaptation to QTP environments. Furthermore, we found that 73.08% (38/52) of the *Rhodococcus* genomes from free-living environments contain [NiFe]-hydrogenase biosynthetic gene clusters, while the remaining eight genomes from endophytes do not (Table S5), underpinning the importance of [NiFe] hydrogenase in coping with harsh or complex environments for *Rhodococcus*. In addition, 17 genes involved in carbon monoxide utilization and three carbonic anhydrase genes are present in the genome of strain FXJ9.536 (Table S4), suggesting its potential to utilize CO and CO_2_ as energy sources [67,68,69,70]. All these data indicate that *R. tibetensis* FXJ9.536 may adapt to nutritional starvation using auxiliary mechanisms for energy production and carbon utilization.

Fourthly, it is reported that some secondary metabolites of rhodococci may have important effects on their growth and environmental adaptation [3]. Hence, the secondary metabolic potential of strain FXJ9.536 was analyzed by antiSMASH. A total of 27 putative sm-BGCs were predicted (Table S6). Three of them showed >30% similarity to known sm-BGCs encoding metabolites with adverse environment adaptation functions. Among them, clusters 1 and 19 showed 57% and 100% similarities to the BGCs of erythrochelin and rhodochelin, respectively, which are two hydroxamate-type siderophores that may chelate extracellular iron from surroundings [71,72]. Cluster 5 showed 42% similarity to the BGC of isorenieratene, a kind of aromatic carotenoid with antioxidant and photoprotective effects [73], and carotenoid production has been linked with fine-tuning membrane fluidity at low temperatures [74]. Thus, these sm-BGCs may also contribute to the survival and reproduction of *R. tibetensis* FXJ9.536 in harsh QTP environments [71,72,73]. Nevertheless, further functional studies are needed to verify the relationship between the sm-BGCs and cold adaptation of strain FXJ9.536.

At last, it is reported that some species within the genus *Rhodococcus* tend to accumulate diverse storage compounds, such as glycogen, polyphosphate, PHAs, and TAGs, which play important roles in environmental adaptions to fluctuating nutritional conditions and metabolic balance [19,20,62,75]. Genes involved in the metabolism of glycogen, polyphosphate, PHAs, and TAGs were also detected in the genome of strain FXJ9.536 (Table S4), indicating that the strain might be able to use these compounds as food storage, so as to thrive in the cold and uninhabited area of the QTP.

### 3.6. Bioinformatics Prospects for Organic Pollutant Degradation Potential of R. tibetensis FXJ9.536

Based on the RAST genomic analysis [45], strain FXJ9.536 was considered to encode genes associated with catabolism of biphenyl/PCBs (eight genes), ethylbenzene (one gene), methane (four genes), alkane (eight genes), and benzoate (eight genes) (Table S3). This result is similar to that of the strain *Rhodococcus* sp. WAY2, which has been reported to be able to degrade multiple organic compounds [76]. According to the genomic information of *R. tibetensis* FXJ9.536, along with its biodegradation activity to 4-NP and malathion, this new species has great potential for environmental remediation against organic pollutants.

## 4. Conclusions

Strain FXJ9.536, isolated from soil in the uninhabited area of the QTP, represents a novel species of *Rhodococcus*, *R. tibetensis* sp. nov. It grows well at 4–28 °C and has good biodegradation activity against 4-NP and malathion. The degradation ratios were 53.6% and 99.9% at 28 °C, and 6.2% and 50.0% at 10 °C, respectively. Genome-based bioinformatics analyses showed that *R. tibetensis* FXJ9.536 possesses the key genes likely responsible for 4-NP and malathion degradation, as well as a number of putative genes for degrading other organic pollutants. Genomic analyses also revealed several potential strategies of this strain to adapt to the harsh QTP environment, i.e., utilizing CSPs, compatible solutes, inorganic compound assimilation systems, secondary metabolites, and storage compounds. Our results indicate that *R. tibetensis* sp. nov. may be a good paradigm for the study of microbial cold adaptation mechanisms and biodegradation of organic pollutants under cold and harsh conditions.

## Data Availability

The data presented in this study are available in the article and supplementary material. The GenBank accession numbers for the 16S rRNA gene and genome sequences of *Rhodococcus* sp. FXJ9.536 are OP167981 and JANFQF000000000, respectively.

## Supplementary Materials

The following supporting information can be downloaded at: https://www.mdpi.com/article/10.3390/microorganisms10101935/s1, Figure S1: Cultural characteristics of strain FXJ9.536 grown on GYM agar for 7 days at different temperatures; Figure S2: HPLC and mass spectrometry (MS) analyses of the degradation residuals of 4-NP in the culture supernatants of strain FXJ9.536; Figure S3: Neighbour-joining phylogenetic tree based on amino acid sequences of the putative 4-hydroxyphenylacetate 3-monooxygenase genes in *Rhodococcus*; Figure S4: GC-MS analysis of the residual malathion in the culture supernatants of strain FXJ9.536; Figure S5: Neighbour-joining phylogenetic tree based on amino acid sequences of the putative carboxylesterase genes in *Rhodococcus*; Figure S6: The biosynthetic gene cluster of Ni/Fe-dependent hydrogenase in the genome of FXJ9.536; Table S1: *Rhodococcus* reference genomes used in this study; Table S2: Genome-wide average nucleotide identities (ANIs) between strain FXJ9.536 and other genome-sequenced *Rhodococcus* strains; Table S3: Genes involved in organic pollutant degradation from strain FXJ9.536; Table S4: Genes involved in stress responses from strain FXJ9.536; Table S5: Numbers of cold shock proteins, trehalose biosynthesis enzymes, and [NiFe] hydrogenase predicted from the genomes of strain FXJ9.536 and other *Rhodococcus* strains; Table S6: Biosynthetic gene clusters of strain FXJ9.536 identified by antiSMASH 5.0.
