# Supplementary material for: Environmental Adaptability and Organic Pollutant Degradation Capacity of a Novel Rhodococcus Species Derived from Soil in the Uninhabited Area of the Qinghai-Tibet Plateau
Source: Microorganisms. 2022 Sep 29;10(10):1935. doi: 10.3390/microorganisms10101935 (PMC9609184; doi:10.3390/microorganisms10101935)
Supplement: Supplementary file 1 [file microorganisms-10-01935-s001.zip › Fig.S_submit.pdf]

Environmental adaptability and organic pollutant degradation capacity of a novel *Rhodococcus* species derived from soil in the uninhabited area of the Qinghai-Tibet Plateau

-- Supplementary materials

Jiao Huang[1, 2], Guomin Ai[1], Ning Liu[1,*], Ying Huang[1, 2,*]

[1]State Key Laboratory of Microbial Resources, Institute of Microbiology, Chinese Academy of Sciences, Beijing 100101, China

[2]College of Life Sciences, University of Chinese Academy of Sciences, Beijing 100049, China

[*]**Correspondence:** Ning Liu, fussliu@126.com; Ying Huang, huangy@im.ac.cn

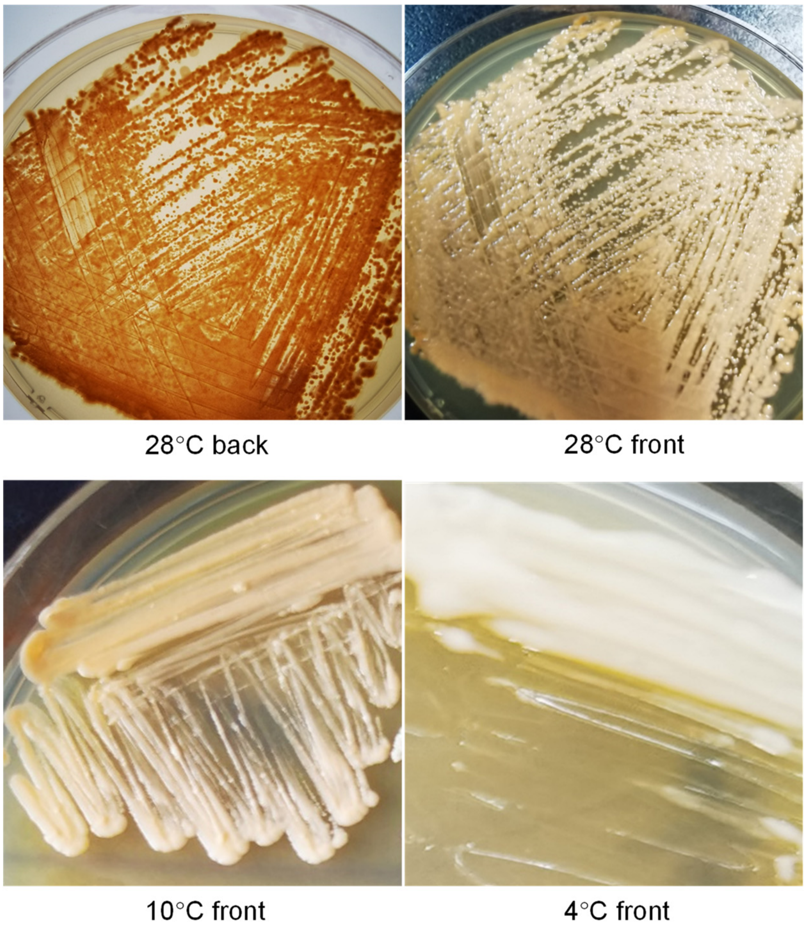

Figure S1. Cultural characteristics of *Rhodococcus tibetensis* FXJ9.536 grown on GYM agar for 7 days at different temperatures.

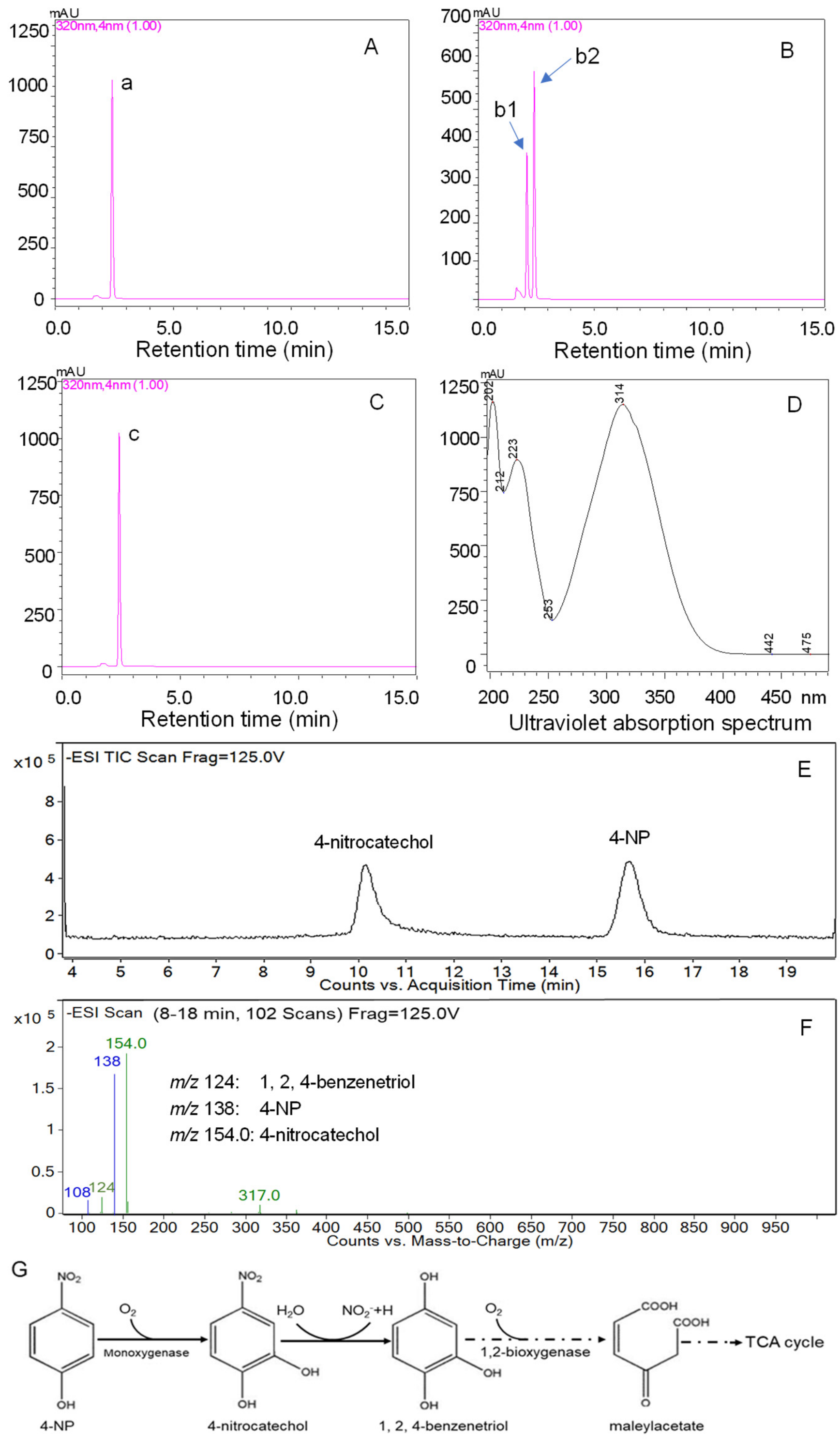

Figure S2. HPLC and mass spectrometry (MS) analyses of the degradation residuals of 4-NP in the culture supernatants of strain FXJ9.536. **A**, HPLC analysis of the uninoculated controls incubated at 28°C and 10°C. **B**, HPLC analysis of the sample incubated at 28°C, where b1 indicates 4-nitrocatechol and b2 indicates 4-NP. **C**, HPLC analysis of the sample incubated at 10°C. **D**, Characteristic ultraviolet absorption spectrum of the peaks a in A, b2 in B, and c in C. **E**, Fine resolution HPLC analysis of the sample incubated at 28°C. **F**, MS analysis of the sample incubated at 28°C. **G**, Proposed biodegradation pathway of 4-NP in strain FXJ9.536.

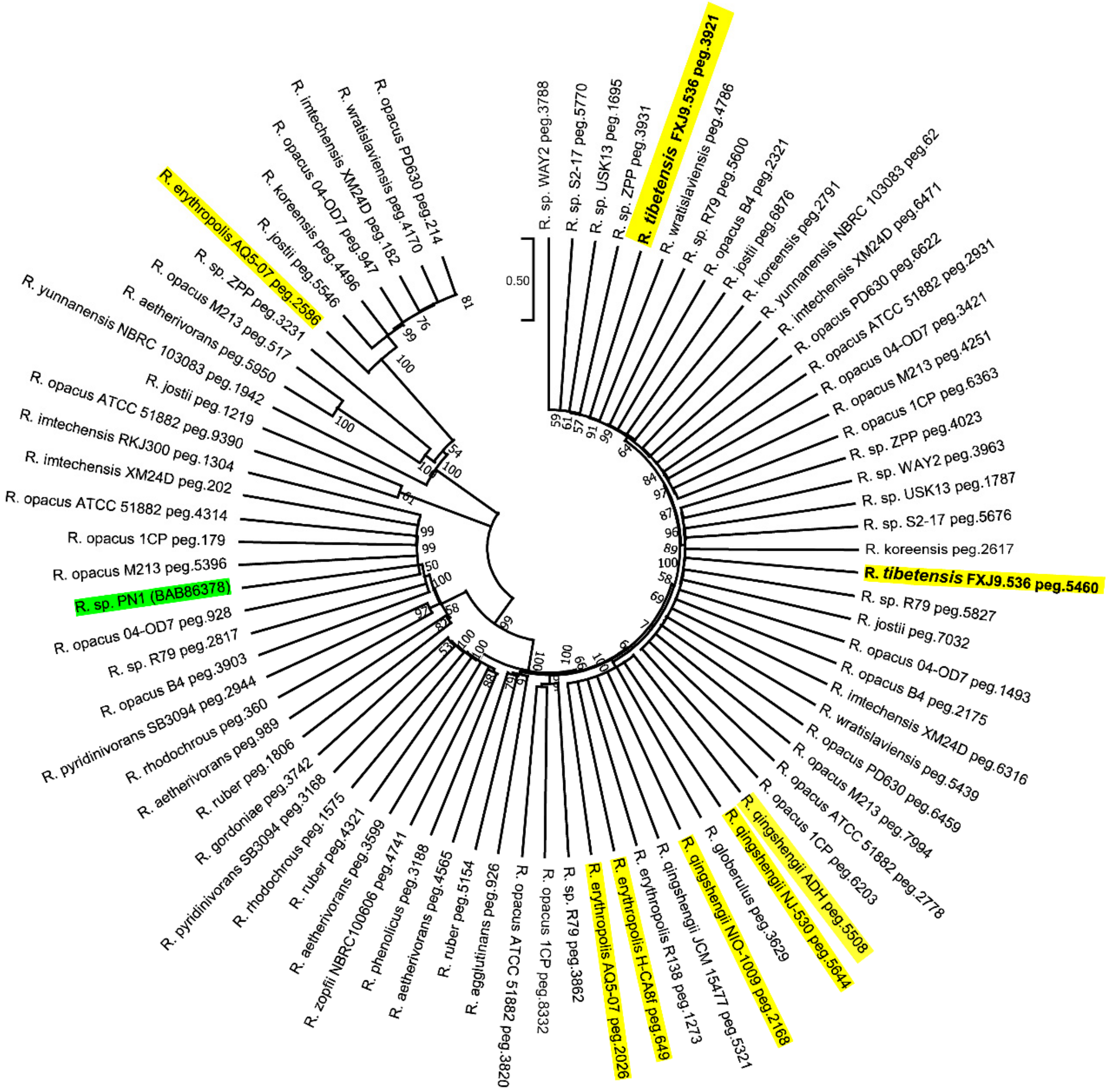

Figure S3. Neighbour-joining phylogenetic tree based on amino acid sequences of the putative 4-hydroxyphenylacetate 3-monooxygenase genes in *Rhodococcus*. Sequences from cold environments and *Rhodococcus* sp. PN1 (4-NP hydroxylase component A) were highlighted in yellow and green, respectively. Bootstrap values (percentages of 1000 replications) above 50% are given. Bar, 0.50 substitutions per site.

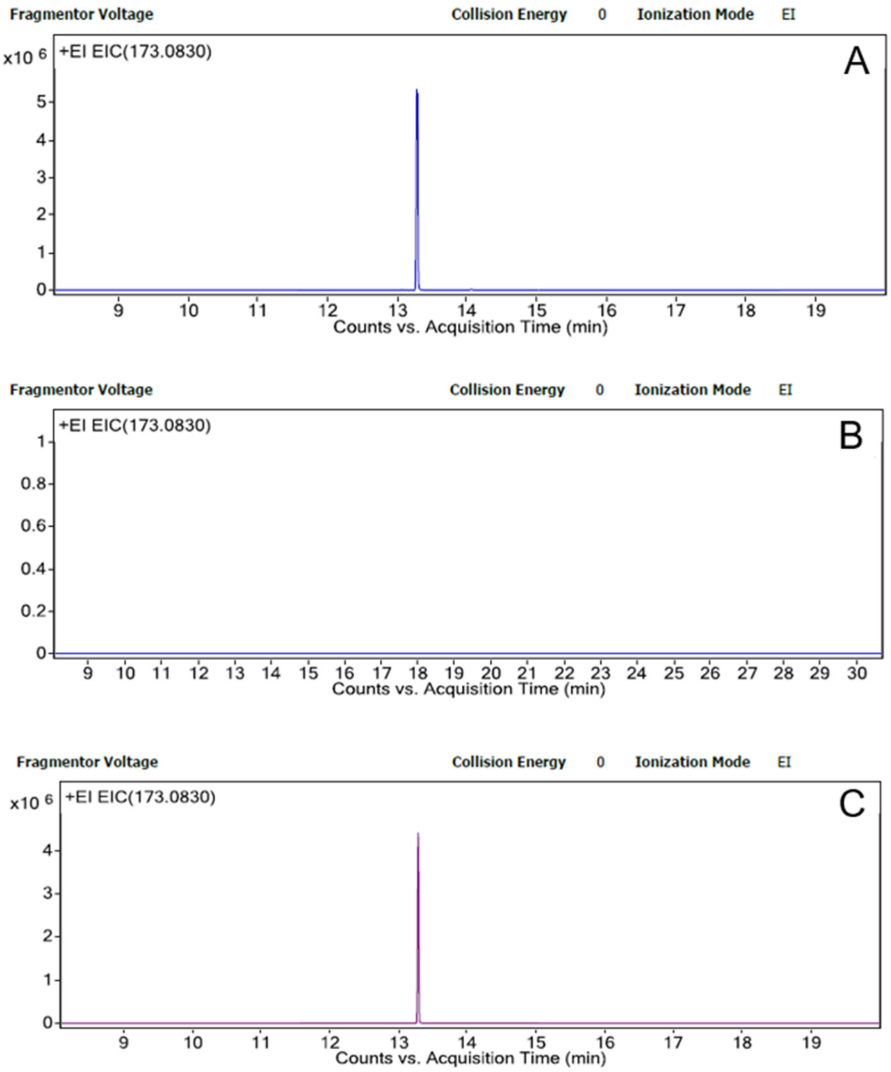

Figure S4. GC-MS analysis of the residual malathion in the culture supernatants of strain FXJ9.536. The figures show the extraction ion chromatogram of *m/z* 173.0830 (±20 ppm), the characteristic fragment ion of malathion. **A**, The uninoculated controls incubated at 24°C and 10°C. **B**, The sample incubated at 24°C. **C**, The sample incubated at 10°C.

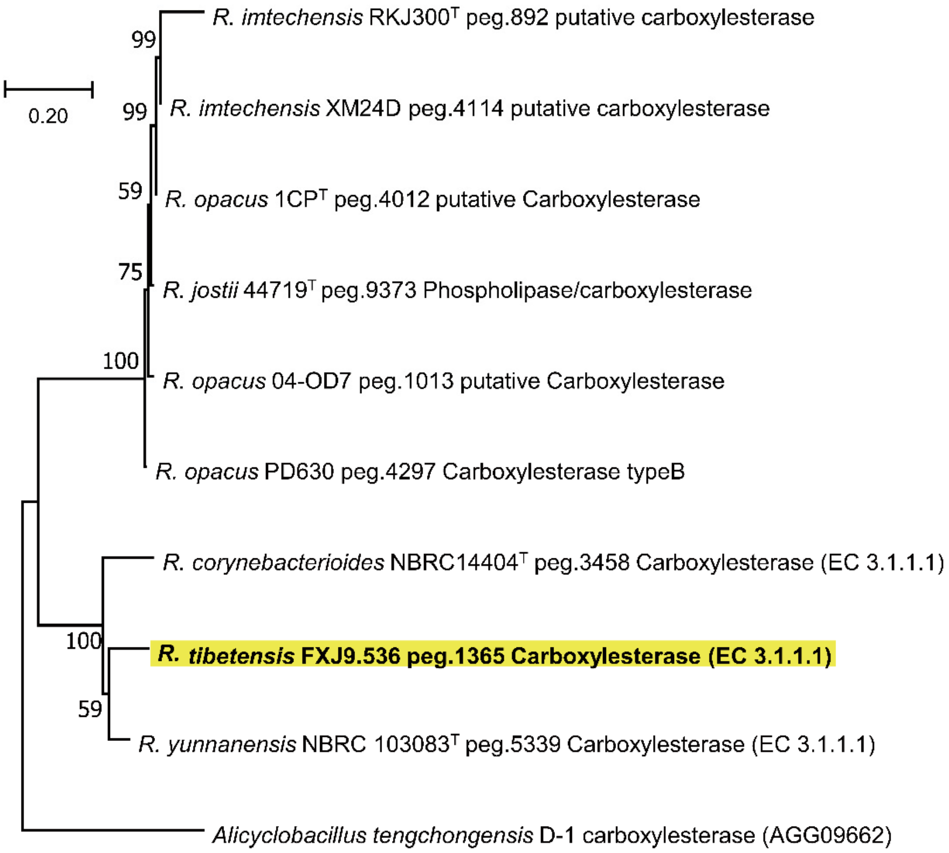

Figure S5. Neighbour-joining phylogenetic tree based on amino acid sequences of the putative carboxylesterase genes in *Rhodococcus*. The carboxylesterase sequence associated with malathion degradation in *Alicyclobacillus tengchongensis* D-1 was used as an outgroup. Bootstrap values (percentages of 1000 replications) above 50 % are given. Bar, 0.20 substitutions per site.

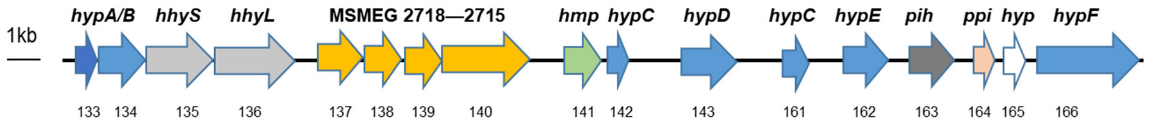

hypA: [NiFe] hydrogenase nickel incorporation protein HypA.

hypB: [NiFe] hydrogenase nickel incorporation-associated protein HypB.

hhyS: [Ni/Fe] hydrogenase, small subunit MSMEG_2720.

hhyL: [Ni/Fe] hydrogenase, large subunit MSMEG_2719.

MSMEG 2718-2715: Uncharacterized NifU-like protein MSMEG_2718,
Uncharacterized protein MSMEG_2717-2715.

hmp: Hydrogenase maturation protease; *hyp*: hypothetical protein.

hypC-E: [NiFe] hydrogenase metallocenter assembly protein HypC-E.

ppi: Phosphoheptose isomerase-like protein 2 found with [NiFe] hydrogenase genes.

hyp: hypothetical protein.

hypF: [NiFe] hydrogenase metallocenter assembly protein HypF.

Figure S6. The biosynthetic gene cluster of Ni/Fe-dependent hydrogenase in the genome of *R. tibetensis* FXJ9.536.
